# A Community Effort to Develop Common Data Elements for Pre-Clinical Spinal Cord Injury Research

**DOI:** 10.1089/neur.2025.0021

**Published:** 2025-04-28

**Authors:** Britt A. Fedor, Abel Torres-Espin, Romana Vavrek, Maryann E. Martone, John L. Bixby, John C. Gensel, Vance Lemmon, Jeffrey S. Grethe, J. Russel Huie, Adam R. Ferguson, Karim Fouad, The Preclinical SCI Common Data Elements (CDE) Workshop Participants, Abel Torres-Espin, Adam Ferguson, Adele Doperalski, Anastasia Keller, Anita Bandrowski, Anushka Sheoran, Britt Fedor, Daryl Fields, David Balser, Deb Hemmerle, Eve Tsai, Fernanda Stapenhorst Franca, George (Chia) Hsu, Jacqueline Bresnahan, Jeffrey S. Grethe, Jiepei Zhu, John Bixby, John Gensel, Karim Fouad, Kazuhiko Kibayashi, Kevin Wang, Kimberly Byrnes, Krithika Iyer, Lex Maliga-Davis, Lingxiao Deng, Lyn Jakeman, Marco Sorani, Maria Bagonis, Maryann Martone, Michael Beattie, Michael Lane, Michelle LaPlaca, Neil Le Belle/Harris, Nikos Kyritsis, Nurul Sulimai, PJ Fairbairn, Preeja Chandran, Rajiv Saigal, Riya Dange, Romana Vavrek Lecerf, Russell Huie, Ruyi Huang, Samir Patel, Samuel Kim, Ubbo Visser, Vance Lemmon, Wolf Tetzlaff, Xuan Duong Fernandez, Zin Khaing

**Affiliations:** ^1^ Faculty of Rehabilitation Medicine, University of Alberta, Edmonton, Canada.; ^2^ Neuroscience and Mental Health Institute, University of Alberta, Edmonton, Canada.; ^3^ School of Public Health Sciences, University of Waterloo, Waterloo, Canada.; ^4^ Department of Neurological Surgery, Brain and Spinal Injury Center (BASIC), Weill Institute for Neurosciences, University of California San Francisco, San Francisco, California, USA.; ^5^ Department of Neurosciences, School of Medicine, University of California San Diego, La Jolla, California, USA.; ^6^ Department of Neurological Surgery, Miami Project to Cure Paralysis and Sylvester Comprehensive Cancer Center, University of Miami Miller School of Medicine, Miami, Florida, USA.; ^7^ Department of Physiology, Spinal Cord and Brain Injury Research Center (SCoBRIC), University of Kentucky College of Medicine, Lexington, Kentucky, USA.; ^8^ San Francisco Veterans Affairs Healthcare System, San Francisco, California, USA.

**Keywords:** common data elements, data management, data sharing, neurotrauma, ODC-SCI, spinal cord injury

## Abstract

For nearly 350 years, the process of disseminating scientific knowledge has remained largely unchanged. Scientists conduct experiments, analyze the data, and publish their findings in the form of scientific articles. Since the turn of the century, this process has been challenged by numerous open science and data sharing efforts to enhance transparency, reproducibility, and replicability of scientific research. Big data approaches, together with machine learning and artificial intelligence, are frequently used to gain insight into the ever-growing complexity of biological systems and biomedical research. To utilize these approaches and harness the continuously increasing computational power requires data to be both machine readable and, ideally, harmonized across studies. Therein lies the challenge: understanding how to organize and describe data is a critical skill for scientists, yet one that is rarely explicitly taught. Common data elements (CDEs), standardized definitions, and reporting structures for data represent a practical solution to this challenge. With the goal of creating a common language to describe and share pre-clinical spinal cord injury (SCI) research data, the open data commons for SCI, in collaboration with the National Institute of Neurological Disorders and Stroke, kicked off this process with the “Preclinical SCI Common Data Elements (CDE) Workshop,” held in conjunction with the National Neurotrauma Symposium in San Francisco, California in June 2024. In this report, we discuss the workshop proceedings, summarize the input provided by the SCI research community, share insights from related CDE efforts, and provide a pragmatic approach to creating CDEs for pre-clinical SCI research.

## Introduction

Today’s researchers create an enormous amount of data. A single biomedical study can produce and collect multiple data types—from basic experimental details, behavioral assays, and histology, to complex physiological monitoring and genomic data. Thus, the volume, variety, and complexity of scientific research data continues to rapidly accelerate.^1^ However, with so much data, it can be a daunting task to tease apart what it all means.

There is little doubt that our ability to manage increasingly large datasets, growing computational power, and the advent of artificial intelligence capable of extracting information inaccessible to the human brain can enhance the predictive value of our data.^1–3^ However, these new opportunities are not without their challenges. As data producers, the value of a dataset as a whole comes from the meaning we ascribe to it. Increasingly, fully realizing the value of our data depends on utilizing computational power—meaning we must organize, manage, and share data in a format that is both human and machine readable.^4^ Furthermore, we need to systematically link the data to meaningful semantic concepts so they can be interpreted.^5^ Such semantic interoperability is often achieved through the creation of structured vocabularies and/or ontologies, that serve to define the classes, properties, and relationships among data entities.^4–7^ It is this link between data and meaning that forms the framework underlying our ability to pool, harmonize, jointly analyze, and interpret the products of different studies.

Data sharing has become prevalent in various research fields over the last two decades, including the field of spinal cord injury (SCI).^8–10^ Yet despite a history of innovation in data sharing, including early efforts to develop publication reporting standards,^2^ and numerous technological and computational advances in recent decades, data sharing in the field of SCI has yet to reach its full potential.^11^ This shortcoming is due, in part, to common and systemic issues found across most scientific disciplines, both those of scientific culture (e.g., a “publish or perish” mentality, incentivizing novelty over replication, flawed funding models, etc.), and those related to data integrity (e.g., reporting bias, lack of reproducibility, and underreporting of research data).^12^ To counteract these issues, efforts to improve data reporting and quality,^13,14^ curation, and sharing^15^ have been gaining momentum. For example, the FAIR Data Principles,^15^ recommendations published in 2016 for making data Findable, Accessible, Interoperable, and Reusable, have been greatly supported by funders. Noteworthy is that many essential data and reporting standards grew out of community-driven efforts.^16^

A prime example of the new age of data transparency is the January 2023 National Institutes of Health (NIH) data management and sharing mandate, which requires researchers to make the data of publicly funded science broadly available.^17^ Similar recommendations and mandates are being made by other funders, and many journals now require the publication of datasets as a condition of article publishing.^18^ Consequently, as the research community moves to a more transparent approach to sharing research, more data sharing platforms are created, putting the value of data publications on the rise.

**FIG. 1.**
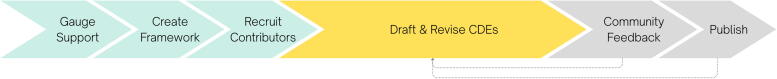
Overview of the CDE development process. Stages are color coded to represent progress in the pre-clinical SCI CDE development initiative: complete (green), in progress (yellow), not started (gray). CDE, common data element; SCI, spinal cord injury.

A guiding initiative in the field of SCI was the development of the Minimum Information About a Spinal Cord Injury experiment (MIASCI) reporting standards, which aimed to improve reporting and promote transparency in published pre-clinical SCI research studies through the adoption of minimum information standards.^2^ It is noteworthy that MIASCI, created in 2014 (nearly a decade before data sharing was mandated), was somewhat ahead of its time. Current adoption of these standards is low and frequently not followed in detail. Yet another step toward data sharing and improved reporting standards in pre-clinical SCI was the establishment of the Open Data Commons for Spinal Cord Injury (ODC-SCI.org),^8–10^ currently the only NIH-recommended data repository for SCI data. This repository addresses FAIR data principles, among others, through a curation process whereby the completeness of both the data and metadata describing the dataset (Dublin Core publishing metadata) and the data themselves (via a data dictionary) are confirmed through expert review and automated checks. To enhance transparency, facilitate data harmonization (the process of unifying different datasets), and simplify analysis of aggregated published data, this curation process evolved to ensure all published datasets include basic required experimental details, termed in the ODC-SCI as the community data elements (CoDEs).^19^ This desire for comparing data derived from different groups, and the evolution of the NIH common data element (CDE) effort in other clinical^20^ and pre-clinical fields^21–23^ led to a collaboration between the ODC-SCI and the NIH to host a kickoff workshop for the creation of CDEs in pre-clinical SCI research, together with the SCI community (Fig. 1).

This report presents the proceedings of the “Preclinical SCI Common Data Elements (CDE) Workshop” held in June 2024 in conjunction with the National Neurotrauma Symposium in San Francisco, California, and organized in collaboration with the National Institute of Neurological Disorders and Stroke (NINDS) and the ODC-SCI. Marking the start of the CDE development process, the workshop engaged members of the research community to discuss (1) the level of support for CDE development and use within the pre-clinical SCI research community, (2) who should be involved in CDE development, and (3) how to structure the CDE development process. Here, we provide a summary of the workshop presentations and the resulting community discussion (see Supplementary Data for workshop agenda) and share community insights on how to best engage and generate buy-in from the wider SCI research community. Using this input, we present a framework for action that outlines how to transform the kickoff workshop’s conceptual discussion of CDEs into a tangible work product to be integrated into our research ecosystem.

### CDEs: What are they?

“A common data element (CDE) is a standardized, precisely defined variable with specific allowable responses for the purpose of enabling interoperability across studies.”*—NINDS*^24^

Simply put, a CDE is a standardized format for reporting, defining, and describing a piece of data (e.g., a single variable or metadata element), that ensures data is consistently collected and reported the same way across different research studies. Through the standardization of variable names, titles, definitions, data types, and permissible values, CDEs define how each data element should be reported and structured (Table 1). Furthermore, CDEs can be developed and structured in such a way as to provide additional context as to the relationships between data elements.^25^ This is often done through the use of classifiers that identify a general data domain (e.g., subject or injury characteristics) or describe importance (e.g., core vs. supplemental), topics discussed in further detail below. Together, this creates a common language to describe research and leaves little room for ambiguity as to what data should be collected or how it should be curated; in turn, this creates a consistent framework for interpreting and reusing data.^25^

**Table 1.** Typical Attributes of Common Data Elements

Term	Description	Example
Data element	A logical unit of data, pertaining to information of one kind	Age of subject
Variable name	A human-readable term that describes the data element	Age
Title	Title of the data element; may be the same as the variable name or expanded to explain abbreviations and/or acronyms used in variable name	Age at time of SCI
Description	A precise definition of the data element that describes what the element is	Age of the subject at the time of SCI, reported in months (calculated as elapsed time between birth and injury)
Unit of measure	Specifies the standard unit of measure applicable to the data element	Months
Permissible values	Specifies accepted values (e.g., pre-defined values or categories) or ranges for the data	0–36
Data type	Specifies the type of data element (e.g., categorical, numeric, ordinal, free text)	Numeric

SCI, spinal cord injury.

Use of CDEs in clinical research has become more common in recent years, largely owing to the efforts of the NINDS CDE Project.^26^ Initiated in 2006, the NINDS CDE Project was developed with the intent to decrease study start-up time, simplify data sharing and aggregation, and facilitate the development of evidence-based guidelines through the creation of data standards for clinical neuroscience research. Early efforts led to the development of the critical core CDE modules, general data standards applicable to all clinical studies regardless of disease and/or disorder (e.g., demographics, medical history, inclusion/exclusion eligibility, adverse events, etc.). Subsequent efforts led to the creation of disease-specific CDEs, with data elements classified as disease core, supplemental-highly recommended, supplemental, or exploratory (Table 2). As of 2024, the NINDS catalog contains >20 disease/disorder-specific CDE sets (e.g., SCI, multiple sclerosis, stroke, and traumatic brain injury [TBI]). However, despite the growing use of CDEs in clinical research—only TBI^21,22^ and post-traumatic epilepsy (PTE)^23^ have established CDEs for pre-clinical neuroscientific data.

**Table 2.** NINDS Recommended CDE Classifications^24^

Classification	Definition
General core	A data element that provides essential information applicable to any study
Disease core	A data element that provides essential information applicable to any study within a given domain
Supplemental—highly recommended	A data element, which is essential based on certain conditions or study type
Supplemental	A data element, which is commonly collected in research studies but whose relevance depends upon the study design or type of research
Exploratory	A data element that requires further validation

CDE, common data element; NINDS, National Institute of Neurological Disorders and Stroke.

### Existing pre-clinical data standards

The MIASCI standards, published in 2014, were established in an effort to improve reproducibility and translational success of basic SCI research.^2^ Noting that experimental design and outcomes vary widely among SCI studies, and most published work does not include sufficient methodological metadata, these standards identified essential information pertaining to 11 common domains of SCI research: investigator, organism, surgery, perturbagen, cell transplantation, biomaterials, histology, immunohistochemistry, imaging, behavior, and data analysis/statistics.^2^ A list of data items and their accepted field type exist for each domain, accompanied by occasional examples or notes that provide further elaboration. Though the MIASCI standards were a great step toward improving research transparency in pre-clinical SCI research, adoption remains low. The work completed by the MIASCI Consortium was then followed by the development of RegenBase, a publicly available knowledge base of SCI biology.^27^ This process resulted in the first machine-understandable ontology to represent experimental SCI research, allowing RegenBase to integrate SCI domain knowledge with data sourced from literature or raw experiments to answer domain-specific questions and aid in the development of data-driven hypotheses.^27^

Recent efforts to enhance transparency and data harmonization in pre-clinical SCI research have been led by the ODC-SCI. In 2022, the ODC-SCI introduced a set of minimum data standards for publication. These standards, referred to as CoDEs,^19^ represent the data elements that must be included and described in a dataset to ensure sufficient metadata exists for data reuse (Table 3). Unlike CDEs, which give a precise definition and permissible values for each variable, CoDEs provide a more general description of what the variable should describe but leave how to describe the details up to the researcher. So, although an accessible starting point toward improving data harmonization, CoDEs leave room for ambiguity in how data are reported and interpreted.^19^ For example, age is described as “age of the subject at start of experiment. If age is available at different timepoints, age is provided at the corresponding time in a corresponding time/timepoint variable.” Researchers may therefore define age using different reference points—some may consider arrival to the animal facility as the start of the experiment, others may consider it the start of pre-injury training, while some may consider it the time of injury. Similarly, without permissible values or more precise detail, age may be defined in days, weeks, months, or years. Though CoDEs are a great improvement, their lack of standardization can cause some confusion and make data harmonization challenging.^19^

**Table 3.** ODC-SCI Community Data Elements

Variable title	Description
Subject ID	Unique identifiers for each subject in the dataset
Species	Species of the subject
Strain	Strain of the subject
Animal origin	Vendor or origin of the animal
Age	Age of the subject at start of experiment. If age is available at different timepoints, age is provided at the corresponding time in a corresponding time/timepoint variable
Weight	Weight of the subject at start of experiment. If weight is available at different timepoints, weight is provided at the corresponding time in a corresponding time/timepoint variable
Sex	Sex of the subject
Group	Name or identifier of the experimental group to which the subject was included if any
Laboratory	Name of laboratory, usually the PI
Study leader	Name of person responsible for overseeing project
Exclusion in origin study	Whether the subject was included in the study that originated the data. “Total exclusion” if excluded from the entire study, otherwise, specify experiment or measures of which the animal was excluded (if any). For example: animals that were run in behavior but maybe tissue is lost and excluded from histological analyses. Reasons for exclusion to be specified in the exclusion reason variable
Exclusion reason	Reason by which the subject was excluded from the study that originated the data as specified in the Exclusion in origin study variable
Cause of death	Cause of death (e.g., perfusion/necropsy, died during surgery, euthanized for health reasons, etc.)
Injury type	Type or model of injury used in the subject (e.g., contusion, complete transaction, partial section)
Injury device	Name of the device used for the injury
Injury level	Spinal cord level at which the injury was performed including segment (e.g., cervical; C) and number (e.g., C5)
Injury details	Other details referent to the injury that might be relevant to understand the severity and type of injury performed

ODC-SCI, open data commons for spinal cord injury.

As the primary goals of creating data standards are to facilitate data collection and improve data harmonization,^20,25,26^ subsequent pre-clinical CDE development initiatives do not need to “reinvent the wheel.” Using a cross-disciplinary approach of adapting established data ontologies, definitions, and conventions from related fields (i.e., pre-clinical TBI and PTE) provides a clear and practical path forward, while also addressing concerns of data integration and interoperability.^28^ For example, the pre-clinical TBI CDE dictionary contains 913 unique CDEs, grouped into three general topics: animal and study metadata, injury models, and assessment and outcomes.^22^ Owing to many similarities in study design, outcomes, and experimental complexity associated with heterogeneity of injury in pre-clinical TBI and SCI, both the organizational framework and the CDEs themselves are likely to overlap. This commonality provides an advantage to those tasked with developing CDEs for pre-clinical SCI, as they will not have to start entirely from scratch to identify, define, and organize relevant data elements.

Previous efforts to create data standards leaned heavily on work completed by other groups and/or in other areas of research. For example, MIASCI leaned heavily on the work produced by the Minimum Information About a Cellular Assay project^29^ and Minimum Information About a Microarray Experiment standards^30^ for guidance on important data elements and developing an ontological framework.

## Community Input

Over the last decade, there has been a noticeable shift within the scientific community, both from researchers and funders, to embrace principles of open science.^31^ In an effort to make science more transparent and reproducible, many researchers freely share information (e.g., standard operating procedures), resources (e.g., transgenic animal lines), and data. While sharing established information or resources is somewhat straightforward, sharing data is often perceived as a time-consuming and onerous task.^32,33^ The creation and adoption of CDEs can alleviate much of the burden of this task; however, it represents a seismic culture shift in how we approach collecting, organizing, maintaining, and sharing data. Therefore, prior to any development efforts, we felt it important to gauge the level of support for development and use of CDEs within the pre-clinical SCI research community (see Supplementary Data for workshop agenda). Breakout sessions were used to facilitate discussion and work toward building community consensus. The goals of the breakout sessions were to (1) assess the level of community support for the use of CDEs, (2) source ideas about how the development process should be structured, (3) identify the overarching domains that describe pre-clinical SCI research, and (4) identify priorities and considerations specific to pre-clinical SCI. Workshop attendees were presented with a list of questions to facilitate discussion (Table 4), then split into breakout groups and asked to document their ideas. The workshop then re-grouped for a general debrief and discussion session. To streamline the process, a representative from each breakout group was chosen to present a discussion summary for one question; all attendees were then given the opportunity to share whether they were in agreement and/or provide other perspectives to be considered. All presentations and discussion sessions were recorded; audio transcripts and discussion notes from the breakout groups were used to summarize the community input into a narrative format.

**Table 4.** Community Discussion Questions

Breakout Session: Would YOU use CDEs? Opinion gathering
1. How do you think CDEs will help your research?
2. Should we start with the CoDEs? What are the pros and cons?
3. Who should be included in the process of CDE creation?
4. How should we organize the process for efficiency and engagement?
5. How do we find and incentivize leadership for the project?
Breakout Session: A path to creating domains for CDEs in pre-clinical SCI
1. What domains present themselves as natural first targets?
2. Are there sub-domains?
3. How do we structure CDE creation—what would a steering committee look like and a domain-specific working group?
4. Who leads these groups?

CDE, common data element; CoDEs, community data elements; SCI, spinal cord injury.

### Gauging support

There was near-unanimous agreement from attendees that CDEs will help their research. Overall, CDEs were thought to be helpful for improving reproducibility and consistency in research. Furthermore, many considered CDEs to be a useful tool to facilitate training and prevent knowledge loss. Understanding what data need to be collected and how it needs to be reported for various frequently used outcomes will aid in prospective experimental planning and ultimately reduce the workload of those responsible for recording and managing data (i.e., trainees and technicians). Additionally, having data organized and described in a standardized format across the field allows for harmonization and comparison of results and/or techniques both within and across labs, and ensures collaborative efforts use a common language. While the structure provided by CDEs was considered beneficial, it was noted that it must be balanced with the need to allow researchers to maintain creativity and innovation in the pre-clinical space.

### Creating buy-in

Discussion of who to engage in the CDE development process revealed that members of the community see development, implementation, and use of CDEs as intertwined elements of the process rather than distinct, sequential steps. Researchers, funders, and other members of the community must be engaged in both the CDE creation process itself *and* in implementing the use of CDEs. Noting that workshop attendees were likely to represent those already in support of creating data standards, tone and messaging were identified as important considerations when advocating for the use of CDEs to those who are unfamiliar with or otherwise unsupportive of their use. It was stressed that CDEs should not be seen as prescriptive, but rather a framework that helps take the guesswork out of deciding what data to collect and how to report it. For example, while recent NIH data sharing mandates mean that researchers must make their data available, little direction has been provided on how to do this from a practical standpoint with respect to everyday lab practice. Rather than simply treating CDEs as a means to an end, educating the community on how CDEs can be used to improve research efficiency, reproducibility, planning, and meet funding requirements was thought to be more beneficial and keep researchers bought into the process for the long term.

Facilitating this transition will require leadership from across the spectrum of research experience. While early-mid career researchers are more likely to share data^34^ and therefore may be more likely to support standards that facilitate this, senior principal investigators (PIs) were considered to be essential in communicating the value of CDEs and equipping trainees with the skills needed to effectively and efficiently use them. By engaging with the CDE development process, researchers of all levels and types of experience have the opportunity to make their voices heard and gain a sense of ownership for their role in guiding the community through this culture shift.

Incentivizing leadership for this project was discussed in less detail. It was generally concluded that promoting this cultural change by educating about the advantages of CDEs in a collaborative effort with the NIH will be the most effective approach. As there will likely only be very limited funding available, involvement should be viewed as community service and based on the interest of researchers. Letting a leadership group evolve naturally and then approaching researchers directly was seen as the best approach to populate committees and workgroups. Indeed, while this article was in development, a steering committee was formed and concerns regarding incentivization did not arise.

### Organizing the process

The community identified the need for inclusivity as a cornerstone of the CDE creation process. Those involved must span disciplines, domains, and experience levels, and include both junior and senior researchers, data scientists, clinician scientists, implementation experts, and persons with lived experience. The varying experience and expertise in this group will allow for a diverse range of perspectives to be heard.

While the process itself must be inclusive, efficiency was a concern. Attendees supported the idea of first forming a small steering committee of five to seven individuals to lead the CDE development initiative and work with support systems (i.e., NIH/NINDS) to create a roadmap for the development process. A major early priority of this steering committee will be to identify three to eight broad domains that encompass the wide variety of experimental models, interventions, and outcomes most frequently used in pre-clinical SCI research. These domains should be identified through a combination of community input (sourced from this workshop) in conjunction with existing CDE domains from the pre-clinical TBI and PTE CDE initiatives. With domains established, the steering committee will then need to engage the wider SCI research community and recruit members for domain-specific working groups. These groups will focus on identifying data elements within their specific domain (e.g., injury characteristics), prioritizing the identification of core (required) data elements.

**FIG. 2.**
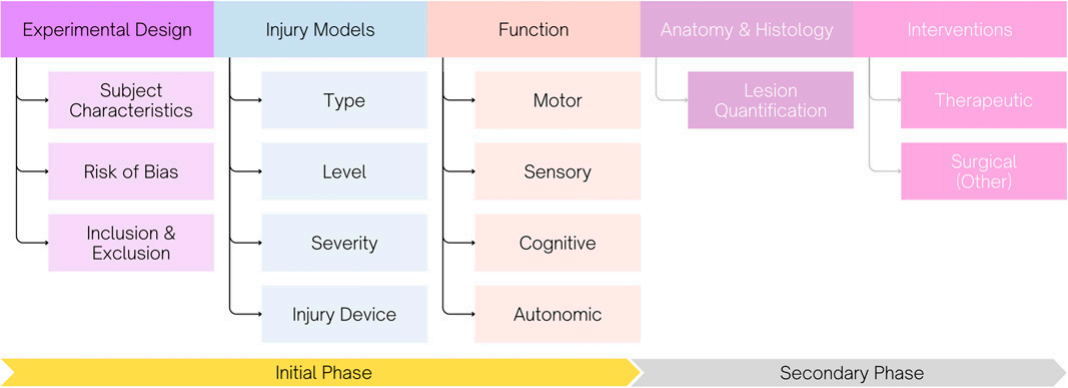
Proposed domains and related sub-domains for pre-clinical SCI CDE development. The initial phase of development will focus on the domains: experimental design, injury models, and function. Each domain will have a dedicated working group, with an additional working group to conduct review and gap analysis of existing pre-clinical CDEs. The anatomy/histology and interventions domains will be tackled in later phases. CDE, common data element; SCI, spinal cord injury.

Rather than taking a bottom-up approach of sifting through all experimental data to identify core variables applicable across the entirety of pre-clinical SCI research, the community advocated for taking a top-down, domain-specific approach informed by work completed in other fields (i.e., TBI^21,22^ and PTE^23^). Several domains presented themselves as natural first targets (Fig. 2), with the community proposing to initiate the CDE development process using data grouped into the following domains:

• Experimental design and risk of bias
• Injury models
• Therapeutic intervention
• Function
• Anatomy and histology

Addressing the volume, variety, and complexity of data contained within these domains will be somewhat challenging. To maximize efficiency, it was suggested that the steering committee identify two to three priority domains for the initial phase of CDE development. As there is substantial overlap between the data collected and reported in pre-clinical TBI, PTE, and SCI, the existing CDEs from these fields should be reviewed. The first working group will be recruited to conduct a gap analysis of the existing TBI and PTE CDEs to determine what already exists and is applicable to SCI and what must be adapted or defined anew. Working in conjunction with the gap analysis group, the domain-specific working groups will be responsible for drafting data elements and classifying them as core (required), highly recommended, or supplemental, with priority in the early phase placed on identifying core data elements.

A general theme emerged from discussion of the ODC-SCI CoDEs and other existing pre-clinical data standards—“don’t reinvent the wheel!” The CoDEs were considered a logical starting point for the development of the initial core pre-clinical SCI CDEs as they represent the “big picture” details required to interpret experimental research. However, the CoDEs in present form leave some room for ambiguity in both interpretation and reporting and therefore require further standardization (e.g., titles, descriptors, permitted values) to ensure data are both precise and harmonizable.

## The Path to Creating (NIH-Endorsed) CDEs: Next Steps

Community discussion of how to structure the CDE development process was generally in alignment with the framework established by NINDS,^26^ an iterative approach that uses the following general strategy:

1. Establish a steering committee and/or working group(s) comprised of experts in the field,
2. Create domain-specific working groups (or identify individuals) to focus on CDE development for each research domain,
3. Draft, review, and refine proposed CDEs,
4. Seek feedback from the wider research community,
5. Incorporate community feedback to refine CDEs,
6. Share CDEs (publish),
7. Incorporate feedback, review, refine, re-share…

**FIG. 3.**
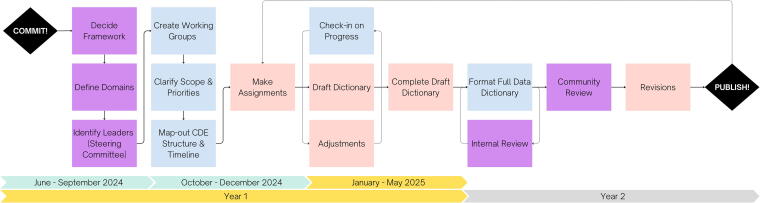
Proposed framework and timeline for the initial phase of the pre-clinical SCI CDE development process. Stages are color coded to represent engaged stakeholder group: SCI research community (purple), CDE steering committee (blue), or domain-specific working groups (pink). As an iterative and collaborative process, stages may be repeated (dotted lines) independently or in sequence before proceeding to the next stage. CDE, common data element; SCI, spinal cord injury.

Based on the level of community support for this initiative, our goal is to have the first set of core pre-clinical SCI CDEs drafted within one year of this meeting (Fig. 3). Three milestones must be achieved to reach this goal: recruitment of the steering committee, identification of priority research domains, and formation of domain-specific working groups. Recruitment of the steering committee is the first logical step, as it will be responsible for overseeing the development process, working with support systems, identifying priority research domains, and inviting members to the working groups.^20^ Once research domains are confirmed and 2–3 priority areas identified, the steering committee will then begin the process of inviting members of the community to the working groups. Aiming to keep inclusion and diversity in mind, recruitment will be informed by type and level of experience as well as area of expertise. In collaboration with the gap analysis group, the domain-specific working groups will identify any critical sub-domains within their area and begin the process of drafting their core CDEs. In an iterative fashion, the steering committee will periodically check-in on the working groups, with adjustments made on an as-needed basis until a draft has been completed for each domain. Following internal review of the drafted CDEs, the steering committee will then initiate the community review process, incorporate feedback and make revisions, and publish the initial pre-clinical SCI CDEs.^26^ A formal process on decision-making has yet to be established. As core data elements from only two to three of the domains will be the priority of this first phase, this process will be repeated and refined as the remaining domains are tackled and as the focus transitions to identifying other classifications of data elements (i.e., highly recommended—supplemental, supplemental, and exploratory). A standardized process for CDE development and structure will be further developed in collaboration with the NINDS and the TBI community.

## Conclusion

In consultation with the SCI research community and experts from related fields,^20–22^ we present a pragmatic framework for the CDE development process that uses and adapts established data ontologies, definitions, and conventions borrowed from related fields. By reporting a common set of variables across all pre-clinical SCI studies, it becomes easier to aggregate large amounts of data, thereby enhancing the predictive value of available data and facilitating the comparison of variables across studies.^1–3^ Furthermore, by borrowing structures and definitions from related fields, such as TBI, we can speed up the development process while also creating an interoperable data ecosystem for pre-clinical neurotrauma that facilitates the comparison of data between often related injuries. While this process signifies a major culture shift in our approach to data, it is a necessary next step toward a future where pre-clinical SCI research data are harmonized, transparent, and communicated in a common language.

## Transparency, Rigor, and Reproducibility Statement

This workshop was conducted under the guidance of the NIH. Zoom was used to record all sessions, and a transcript of the audio was generated. The audio transcript was reviewed (R.V.) for accuracy and completeness, and the video recording was consulted if concerns regarding accuracy or completeness of the transcript arose (i.e., incomplete sentences or abrupt transitions). Meeting proceedings were summarized as presented in this article (B.A.F.; K.F.) and approved by all authors.

## Abbreviations Used

CDE: Common Data Element
CoDEs: Community Data Elements
FAIR: Findable, Accessible, Interoperable, Reusable
MIASCI: Minimum Information About a Spinal Cord Injury experiment
NIH: National Institutes of Health
NINDS: National Institute of Neurological Disorders and Stroke
PI: Principal Investigator
PTE: Post-traumatic Epilepsy
ODC-SCI: Open Data Commons for Spinal Cord Injury
SCI: Spinal Cord Injury
TBI: Traumatic Brain Injury

## Appendix A1. Pre-Clinical SCI Common Data Elements (CDE) Workshop Participants

Abel Torres-Espin, Adam Ferguson, Adele Doperalski, Anastasia Keller, Anita Bandrowski, Anushka Sheoran, Britt Fedor, Daryl Fields, David Balser, Deb Hemmerle, Eve Tsai, Fernanda Stapenhorst Franca, George (Chia) Hsu, Jacqueline Bresnahan, Jeffrey S. Grethe, Jiepei Zhu, John Bixby, John Gensel, Karim Fouad, Kazuhiko Kibayashi, Kevin Wang, Kimberly Byrnes, Krithika Iyer, Lex Maliga-Davis, Lingxiao Deng, Lyn Jakeman, Marco Sorani, Maria Bagonis, Maryann Martone, Michael Beattie, Michael Lane, Michelle LaPlaca, Neil Le Belle/Harris, Nikos Kyritsis, Nurul Sulimai, PJ Fairbairn, Preeja Chandran, Rajiv Saigal, Riya Dange, Romana Vavrek Lecerf, Russell Huie, Ruyi Huang, Samir Patel, Samuel Kim, Ubbo Visser, Vance Lemmon, Wolf Tetzlaff, Xuan Duong Fernandez, and Zin Khaing.
