# Supplementary material for: A Community Effort to Develop Common Data Elements for Pre-Clinical Spinal Cord Injury Research
Source: Neurotrauma Rep. 2025 Apr 28;6(1):391–401. doi: 10.1089/neur.2025.0021 (PMC12408885; doi:10.1089/neur.2025.0021)
Supplement: Supplementary Data [file neur.2025.0021_supplementary_data.docx]

**Preclinical SCI Common Data Elements CDE Workshop Agenda**

June 8-9, 2024

Hilton San Francisco Union Square Hotel, San Francisco, CA

**Day I: Saturday, June 8**

8:00-8:30 **Welcome and introduction and history of CDEs**

The path to creating (NIH-endorsed) CDEs in preclinical SCI

*(Presenter: Lyn Jakeman)*

8:30-9:00 **Defining CDEs and why are they useful**

Example clinical SCI CDEs

*(Presenters: John Gensel/Xuan Duong Fernandez)*

9:00-9:30 **ODC community elements (CoDEs)**

*(Presenters: Anushka Sheoran/Abel Torres-Espin/Maryann Martone)*

9:45-10:15 **Coffee break**

10:15-10:45 **CDEs in preclinical TBI research: what are the lessons learned?**

*(Presenters: Michelle LaPlaca/Neil Harris)*

10:45-12:00 **Breakout session 1**

Would YOU use CDEs? Opinion gathering

*(Moderators: Lyn Jakeman/Adam Ferguson)*

12:00-13:00 **Working lunch**

Team to present questions to the TBI audience about the TBI experience and lessons learned during the CDE creation process

**Preclinical SCI Common Data Elements CDE Workshop Agenda**

June 8-9, 2024

Hilton San Francisco Union Square Hotel, San Francisco, CA

**Day II: Sunday, June 9**

8:00-8:05 **Review Day II agenda**

8:05-8:30 **The view toward the SCI community**

A brief conversation between a nurse-scientist, and neurodata expert with lived experience about quality-of-life concerns in human SCI and how they relate to CDEs

*(Presenters: Marco Sorani, PhD/Debra Hemmerle, RN, PhD)*

*(Moderator: Adam Ferguson)*

8:30-9:00 **Domains for CDEs in preclinical SCI**

*(Presenters: Vance Lemmon/John Bixby/Jeff Grethe/Ubbo Visser)*

9:00-9:30 **Let’s discuss the first impressions: What are you thinking?**

*(Moderator: Karim Fouad)*

9:30-11:00 **Breakout session 2**

A path to creating domains for CDEs in preclinical SCI

*(Moderator: Karim Fouad*)

11:00-11:30 **Read out from working Groups**

*(Moderator: Lyn Jakeman)*

11:30-12:00 **Conclusion and volunteer recruitment for future work groups**

*(Moderators: Adam Ferguson/John Gensel)*
